# Modulation of Macrophage Activation State Protects Tissue from Necrosis during Critical Limb Ischemia in Thrombospondin-1-Deficient Mice

**DOI:** 10.1371/journal.pone.0003950

**Published:** 2008-12-16

**Authors:** Nicolas Bréchot, Elisa Gomez, Marine Bignon, Jamila Khallou-Laschet, Michael Dussiot, Aurélie Cazes, Cécile Alanio-Bréchot, Mélanie Durand, Josette Philippe, Jean-Sébastien Silvestre, Nico Van Rooijen, Pierre Corvol, Antonino Nicoletti, Bénédicte Chazaud, Stéphane Germain

**Affiliations:** 1 INSERM, U833, Paris, France; 2 Laboratoire Angiogenèse Embryonnaire et Pathologique, Collège de France, Paris, France; 3 INSERM, U872, Paris, France; 4 Centre de recherche des Cordeliers, Université Pierre et Marie Curie, Paris, France; 5 Laboratoire d'Hématologie, Hôpital Bicêtre, AP-HP, Le Kremlin-Bicêtre, France; 6 INSERM, U689, Paris, France; 7 Centre de Recherche Cardiovasculaire de Lariboisière, Université Denis Diderot, Paris, France; 8 Department of Molecular Cell Biology, Free University Medical Center, Amsterdam, The Netherlands; 9 INSERM, U 567, CNRS UMR 8104, Paris, France; 10 Institut Cochin, Université Paris 5, Paris, France; 11 Service d'Hématologie Biologique A, Hôpital Européen Georges Pompidou, AP-HP, Paris, France; Karolinska Institutet, Sweden

## Abstract

**Background:**

Macrophages, key regulators of healing/regeneration processes, strongly infiltrate ischemic tissues from patients suffering from critical limb ischemia (CLI). However pro-inflammatory markers correlate with disease progression and risk of amputation, suggesting that modulating macrophage activation state might be beneficial. We previously reported that thrombospondin-1 (TSP-1) is highly expressed in ischemic tissues during CLI in humans. TSP-1 is a matricellular protein that displays well-known angiostatic properties in cancer, and regulates inflammation *in vivo* and macrophages properties *in vitro*. We therefore sought to investigate its function in a mouse model of CLI.

**Methods and Findings:**

Using a genetic model of *tsp-1* ^−/−^ mice subjected to femoral artery excision, we report that *tsp-1* ^−/−^ mice were clinically and histologically protected from necrosis compared to controls. Tissue protection was associated with increased postischemic angiogenesis and muscle regeneration. We next showed that macrophages present in ischemic tissues exhibited distinct phenotypes in *tsp-1* ^−/−^ and *wt* mice. A strong reduction of necrotic myofibers phagocytosis was observed in *tsp-1* ^−/−^ mice. We next demonstrated that phagocytosis of muscle cell debris is a potent pro-inflammatory signal for macrophages *in vitro*. Consistently with these findings, macrophages that infiltrated ischemic tissues exhibited a reduced postischemic pro-inflammatory activation state in *tsp-1* ^−/−^ mice, characterized by a reduced Ly-6C expression and a less pro-inflammatory cytokine expression profile. Finally, we showed that monocyte depletion reversed clinical and histological protection from necrosis observed in *tsp-1* ^−/−^ mice, thereby demonstrating that macrophages mediated tissue protection in these mice.

**Conclusion:**

This study defines targeting postischemic macrophage activation state as a new potential therapeutic approach to protect tissues from necrosis and promote tissue repair during CLI. Furthermore, our data suggest that phagocytosis plays a crucial role in promoting a deleterious intra-tissular pro-inflammatory macrophage activation state during critical injuries. Finally, our results describe TSP-1 as a new relevant physiological target during critical leg ischemia.

## Introduction

Peripheral artery disease affects up to 15% of people over 55 years [1] and may lead to critical limb ischemia (CLI) as the disease progresses. Despite percutaneous transluminal angioplasty or vascular surgery, major amputation occurs in about 13% of patients suffering from CLI [2], thereby emphasizing the crucial need for alternative efficient pharmacological treatments. Several studies in humans were designed with the aim of restoring proangiogenic signals in ischemic legs, but led to contradictory results [3]–[5]. Physiopathology of CLI is indeed complex and not restricted to solely a lack of tissue perfusion. Inflammation is also a crucial component of critical leg ischemia in humans since ischemic tissues exhibit large inflammatory infiltrates, rich in macrophages [6], [7], which are known to be key regulators of healing/regeneration processes [8]–[10]. However pro-inflammatory markers independently correlate with disease progression (relative risk = 2.9), risk of amputation and 1-year mortality [11], suggesting a potential deleterious effect of the pro-inflammatory state observed in patients.

Thrombospondin-1 (TSP-1) is a 450 kDa matricellular protein synthesized by various cell types, that interacts with a wide range of integrin and non-integrin receptors, thus exhibiting pleiotropic activities [12]. It accumulates during various situations of tissue injury, and acts as a regulator of tissue remodeling [13], [14]. In particular it displays potent angiostatic properties in cancer [14], [15] and limits ischemic tissue survival in a myocutaneous flap model through inhibition of NO-mediated post-ischemic vasorelaxation [16]. Moreover TSP-1 is a key regulator of inflammation in mice *in vivo* [17], [18], and is a strong regulator of macrophage properties *in vitro*. It is a potent pro-inflammatory [19] and pro-migratory [20] signal for macrophages, and phagocytosis of various cell types depends on TSP-1 [21]–[25].

We previously showed that TSP-1 is strongly overexpressed during critical hind limb ischemia in humans and secreted by both endothelial cells and macrophages [26]. Considering its complex roles in regulating both angiogenesis and inflammation, we here hypothesized that TSP-1 may play a deleterious role in CLI, and therefore sought to investigate its function in a mouse model of CLI. We report that *tsp-1* ^−/−^ mice were clinically and histologically protected from tissue necrosis induced by limb ischemia. Tissue protection was associated with increased postischemic angiogenesis and muscle regeneration. We next showed that macrophages in ischemic tissues exhibited distinct phenotypes in *tsp-1* ^−/−^ and *wt* mice: phagocytosis of necrotic myofibers was strongly reduced in *tsp-1* ^−/−^ mice. Consistently with our findings that phagocytosis of muscle cell debris is a potent pro-inflammatory signal, macrophages exhibited a reduced pro-inflammatory activation state in these mice. Finally, using a model of monocyte depletion, we demonstrated that this distinct macrophage phenotype was responsible for the tissue protection observed in *tsp-1* ^−/−^ mice.

## Results

### TSP-1 is expressed by macrophages and endothelial cells during critical hind limb ischemia in mice

**Figure 1:**
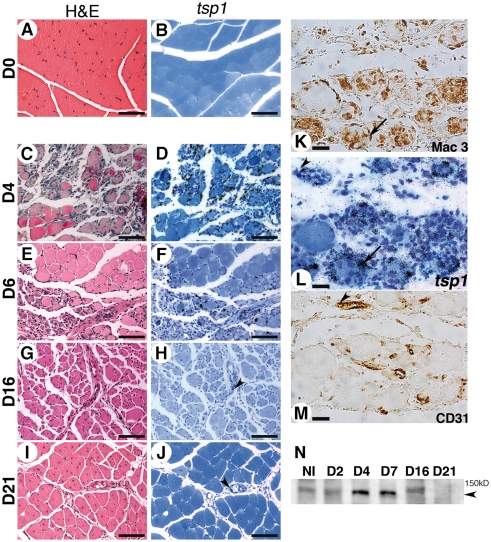
Thrombospondin-1 is expressed during critical hind limb ischemia in mice. Thrombospondin-1 mRNA expression was analyzed in gastrocnemius muscle using in situ hybridization at d4 (D), d6 (F), d16 (H) and d21 (J) after ischemia. D0 (B) represents non ischemic tissue. Adjacent sections (respectively C, E, G, I and A were stained with H&E (Scale bar = 200 µm). (L) Gastrocnemius muscle analyzed at d4 for tsp-1 mRNA expression at a higher magnification (scale bar = 25 µm). (K, M) Adjacent sections were stained for macrophages (K) and endothelial cells (M), showing expression of tsp-1 mRNA in macrophages (see arrows in K&L) and endothelial cells (see arrowheads in L&M). Arrowheads in H&J show tsp-1 mRNA expression in endothelial cells at d16 and d21 respectively. (N) Western blot analysis of TSP-1 expression in gastrocnemius muscle at similar time points. Arrowhead = 150 kD.

We analyzed *tsp-1* mRNA expression pattern in gastrocnemius muscle during CLI in mice at d4, d6, d16 and d21 following femoral artery excision. No expression was observed in non ischemic tissues (fig 1A&B). At early time points (d4), tissue architecture was strongly disorganized in necrotic areas (fig. 1C & fig. S1A), replaced by an inflammatory infiltrate rich in macrophages (fig. S1B). A dense network of capillaries developed at this stage (fig. S1C), not covered by smooth muscle cells (not shown). *Thrombospondin-1* mRNA was highly expressed in necrotic areas (fig. 1D), expressed by macrophages (fig. 1K&1L), endothelial cells (fig. 1L&1M), and to a lesser extent by myofibers (fig. 1D). In contrast to heart or brain [9], [27], skeletal muscle displays regenerative properties during ischemia [28]. At d6 regenerating basic myofibers appeared in the healing area (fig. 1E & fig. S1D). At this stage, *tsp-1* mRNA was still expressed by macrophages localized in the healing border zone (fig. 1F & fig. S1E). From d16 to d21, regenerating myofibers with central nuclei developed (fig. 1G & fig. S1G) and gastrocnemius muscle healed almost normally, except small lipidic deposits observed locally (fig. 1I & fig. S1J). Macrophages gradually disappeared (fig. S1H&1K) at these later stages and *tsp-1* mRNA expression strongly decreased, nevertheless persisting in endothelial cells (see arrowheads fig. 1H&1J). As previously described [28], capillary density decreased during the regeneration process (fig. S1C-1L).

Western blot analysis of TSP-1 protein expression at similar time points confirmed protein expression in muscles, that peaked at early stages (d4 to d7) and then decreased at d16 until d21 (fig. 1N).

We thus found that TSP-1 is expressed during postischemic healing/regeneration in macrophages and endothelial cells in mice, as we previously observed in humans [26].

### *Thrombospondin-1* ^−/−^ mice are protected from ischemia-induced necrosis

**Figure 2:**
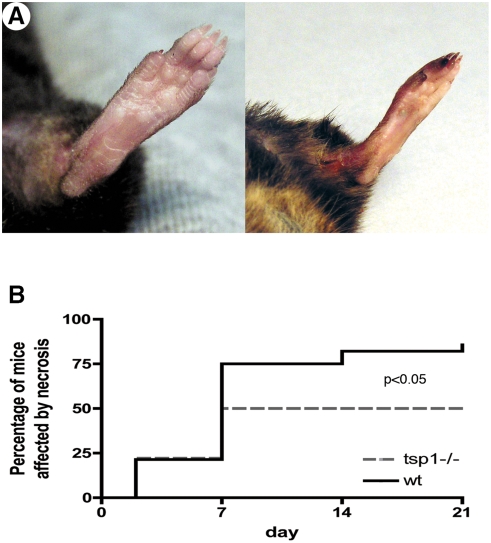
Clinical necrosis is reduced in tsp-1−/− mice. (A) Pictures show mice either protected from necrosis (left) or affected by necrosis (right). (B) Cumulative incidence of necrosis was followed during 21 days after ischemia in both wt and tsp-1−/− mice and represented as Kaplan-Meier estimates (n = 19; p<0.05 tsp-1−/− vs. wt).

In order to analyze the functional role of TSP-1 during critical hind limb ischemia, we performed femoral artery excision in *tsp-1* ^−/−^ mice and their *wt* littermates and followed macroscopic clinical necrosis during 21 days. Figure 2A shows two representative pictures of either tissue protection or necrosis. Necrosis developed between d2 and d7 (fig. 2B). *Wild-type* mice were highly affected since 86% of mice exhibited macroscopic necrosis (fig. 2B). Conversely, only 50% of *tsp-1* ^−/−^ mice were affected (p<0,05 *vs. wt*, n = 19).

**Figure 3:**
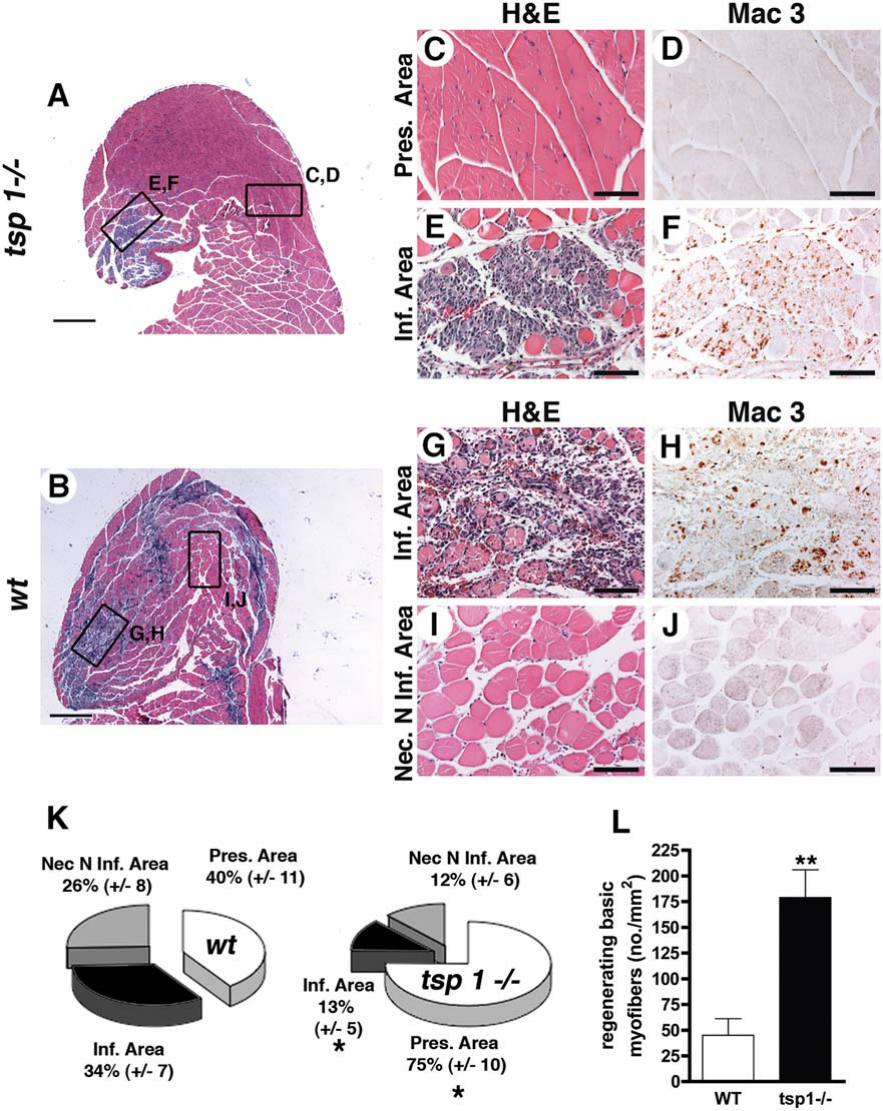
Histological analysis showing tissue protection in tsp-1−/− mice. (A&B) H&E staining of gastrocnemius muscle sections at d4 were performed in tsp-1−/− (A) and wt (B) mice (scale bar = 500 µm). Right panel show higher magnifications of preserved area (C), infiltrated area in tsp-1−/− (E) and in wt (G) and necrotic non-infiltrated area (I), with adjacent slides immunostained for macrophages using Mac-3 Ab (D, F, H, J, respectively); scale bar = 100 µm. (K) Quantification of histologically different area types surface (see text for definition); mean±S.E.M. are shown, * = p<0.05, n = 9. (L) Quantification of regenerating basic myofibers in necrotic areas, mean±S.E.M. are shown, ** = p<0.01, n = 9.

We then performed histological analyses of gastrocnemius muscles from *tsp-1* ^−/−^ and *wt* mice at d4 (d4 was chosen because necrosis is an ongoing process at this time point (fig. 2B)). Tissue protection was confirmed in *tsp-1* ^−/−^ mice (fig. 3). Three different types of area were observed at this stage as described in the methods section and in fig. S2 : i) a preserved area presenting an almost normal histology (fig. 3A&C–D); ii) an infiltrated area, in which necrotic tissue was replaced by a cell infiltrate rich in macrophages (fig. 3A&E–F; fig. 3B&G–H); iii) a necrotic non-infiltrated area, which contained necrotic myofibers exhibiting a pale eosinophilic cytoplasm with oedema and a loss of peripheral nuclei [29], [30]) (fig. 3B&I–J). The surface of each area type was quantified in each mouse and reported as a percentage of the entire histological section surface (fig. 3K). Preserved area was strongly increased in *tsp-1* ^−/−^ mice. In parallel, a significantly reduced necrotic infiltrated area surface was observed. Finally, *tsp-1* ^−/−^ mice exhibited a reduced necrotic non-infiltrated area, though not reaching statistical significance (fig. 3K). We next quantified regenerating basic myofibers in both genotypes and showed that tissue regeneration was also strongly improved in *tsp-1* ^−/−^ mice (fig. 3E&3G and fig. 3L). Using both clinical and histological evaluation, we thus demonstrated a strong tissue protection from necrosis in *tsp-1* ^−/−^ mice during CLI.

### Postischemic angiogenesis is increased in *tsp-1* ^−/−^ mice

**Figure 4:**
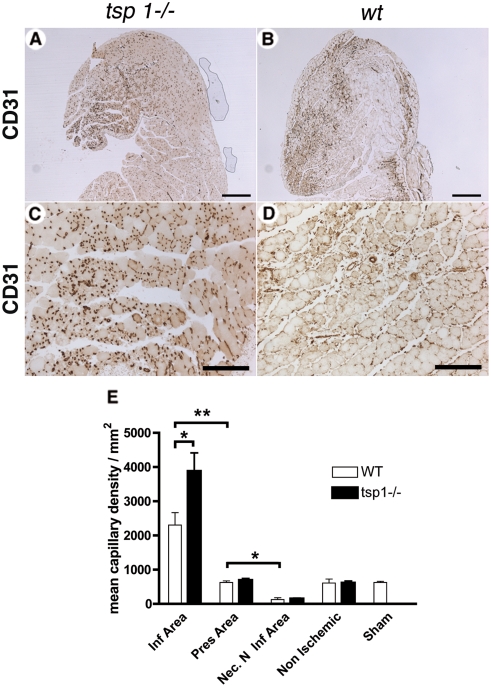
Postischemic angiogenesis is increased in tsp-1−/− mice. (A&B) Capillary density of gastrocnemius muscle sections was assessed at d4 using CD31 immunostaining in tsp-1−/− (A) and wt (B) mice (scale bar = 500 µm). (C&D) CD31 immunostaining of infiltrated area in tsp-1−/− (C) and wt (D) mice; scale bar = 200 µm. (E) Quantification of capillary density in the different area types in gastrocnemius muscles, d4 after ischemia. Mean±SEM are shown. * = p<0.05; ** = p<0.01; n = 9.

We next sought to analyze whether angiogenesis was modulated at d4 in the three areas described above in *tsp-1* ^−/−^ and *wt* mice by assessing capillary density in gastrocnemius muscles using CD31 immunostaining (fig. 4A–D). In *wt* mice, capillary density increased only in the infiltrated area, whereas it remained similar to non ischemic tissue in the preserved area and decreased in the necrotic non-infiltrated area (fig. 4E). In *tsp-1* ^−/−^ mice, postischemic capillary density was further increased in the infiltrated area compared to *wt* mice (fig. 4E), whereas no difference was found between both genotypes in areas which were not infiltrated by macrophages, as well as in non ischemic tissues.

Mice also underwent limb arteriographies at d4 and images of vessel networks were quantified in order to assess arteriogenesis. No difference was observed between both genotypes (not shown). Anti-α-smooth muscle actin immunoanalysis also revealed the same density of covered vessels (not shown).

Taken together, these results show that *tsp-1* ^−/−^ mice exhibited an increased postischemic angiogenesis, which was limited to capillary formation at d4 and was restricted to areas infiltrated by macrophages.

### *Thrombospondin-1* ^−/−^ macrophages exhibit a reduced phagocytotic ability *in vivo* and *in vitro*

**Figure 5:**
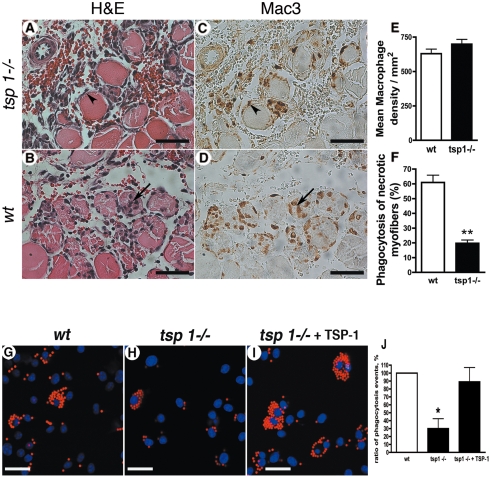
Postischemic angiogenesis is increased in tsp-1−/− mice. (A&B) Capillary density of gastrocnemius muscle sections was assessed at d4 using CD31 immunostaining in tsp-1−/− (A) and wt (B) mice (scale bar = 500 µm). (C&D) CD31 immunostaining of infiltrated area in tsp-1−/− (C) and wt (D) mice; scale bar = 200 µm. (E) Quantification of capillary density in the different area types in gastrocnemius muscles, d4 after ischemia. Mean±SEM are shown. * = p<0.05; ** = p<0.01; n = 9.

As macrophages play a key role during healing/regeneration processes [8], [9] and angiogenesis [31], express *tsp-1* and are described as a potential TSP-1 target [19], we further sought to investigate the potential differential role of macrophages in both strains. Gastrocnemius muscles at the same stage of regeneration were immunostained for macrophages (fig. 5A–5D). Macrophage density was identical in *tsp-1* ^−/−^ and *wt* mice (fig. 5E), thereby ruling out a major effect of TSP-1 on monocyte recruitment. However, macrophages localized differently in the infiltrated area in both groups: in *wt* mice, macrophages had a widespread distribution, mostly into myofibers undergoing phagocytosis (fig. 5B&5D). In contrast, macrophages remained mostly outside of myofibers in *tsp-1* ^−/−^ mice (fig. 5A&5C). When quantified, phagocytosis of necrotic myofibers was strongly reduced in *tsp-1* ^−/−^ mice (fig. 5F).

We then analyzed *in vitro* phagocytotic properties of isolated bone marrow derived macrophages from both genotypes using fluorescent latex beads (fig. 5G–J). Our results show that *tsp-1* ^−/−^ macrophages had a reduced ability to engulf beads *in vitro* compared to *wt* macrophages (fig. 5G&5H). In addition, this property could be rescued by adding recombinant TSP-1 in the culture medium (fig. 5I).

These data indicate a strong impairment of phagocytotic ability in *tsp-1* ^−/−^ macrophages, *in vitro* and *in vivo* during CLI.

### Phagocytosis of necrotic muscle cells debris is a strong pro-inflammatory signal for macrophages

**Figure 6:**
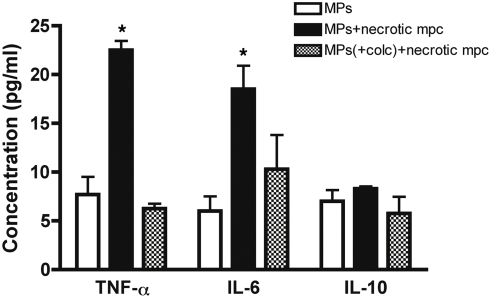
Phagocytosis of necrotic muscle cells debris is a pro-inflammatory signal for macrophages. TNF-α, IL-6 and IL-10 secretion was quantified by ELISA in the conditioned medium of IFN-γ-activated macrophages, pre-treated or not with 10 µg/ml Colchicine (+colc) for 30 min, after phagocytosis of necrotic myogenic precursor cells (mpc). Mean±SEM of three experiments are shown. * = p<0,05 vs. control.

We next sought to analyze the effect of phagocytosis of muscle cells debris on the cytokine expression profile of macrophages. Upon phagocytosis of necrotic myogenic precursor cells (mpc), TNF-α and IL-6 secretion strongly increased, whereas IL-10 secretion remained unchanged (fig. 6). When macrophages were treated with Colchicine to inhibit phagocytosis [32], TNF-α and IL-6 induced secretion was abolished in the presence of mpc debris (fig. 6). These data demonstrate that phagocytosis of muscle cells debris is a pro-inflammatory signal for macrophages.

### *Thrombospondin-1^−/−^* mice exhibit a less pro-inflammatory macrophage activation state in response to ischemia

We therefore next investigated a potential different activation state of macrophages in response to ischemia in both genotypes. We first characterized intra-tissular macrophage activation state at various time points after ischemia in C57Bl6 *wt* mice (fig. S3A), by analyzing macrophages using Fluorescence-Activated Cell Sorting (FACS) for F4/80 and Ly-6C expression, a membrane marker of macrophage pro-inflammatory activation state [8], [9], [33], [34]. Primary invading cells consisted in an homogeneous population of SSC(lo) CD11b(hi) macrophages (fig. S3B and [33]) that exhibited a F4/80(lo)Ly-6C(hi) membrane marker expression profile. At later time points, a transition from F4/80(lo)Ly-6C(hi) to F4/80(hi)Ly-6C(lo) macrophages was observed (fig. S3A).

**Figure 7:**
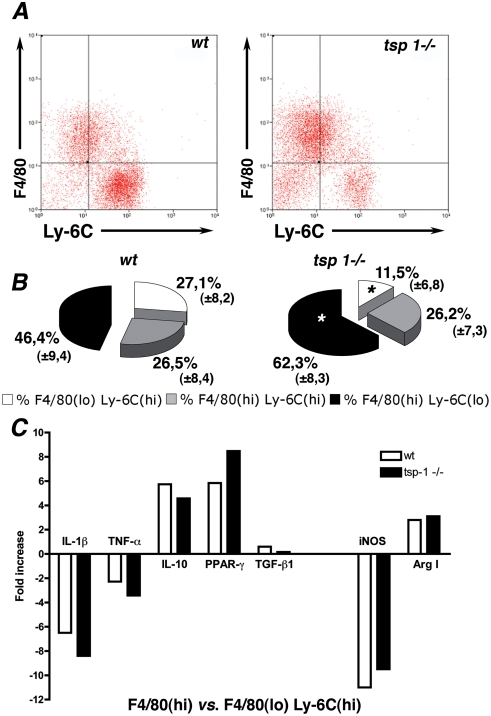
Thrombospondin-1−/− mice exhibit a less pro-inflammatory macrophage activation state in response to ischemia. (A) FACS analyses of CD45+ cells isolated from ischemic muscles at d4, stained for F4/80 and Ly-6C expression, wt, left panel; tsp-1−/−, right panel; n = 5 mice per group. (B) Quantification of F4/80(lo)Ly-6C(hi), F4/80(hi)Ly-6C(hi) and F4/80(hi)Ly-6C(lo) macrophage proportions in CD45+ cells isolated from ischemic muscles at d4 in both genotypes (n = 5 mice per group in five independent experiments). * = p<0,05 vs. wt. (C) Quantitative RT-PCR analyses of IL-1β, TNF-α, IL-10, PPAR-γ, TGF-β, iNOS and Arg1 expression in F4/80(hi) vs. F4/80(lo)Ly-6C(hi) cells isolated from ischemic muscles at d4 in both genotypes.

We next investigated macrophage activation state in both genotypes. CD 45+ inflammatory cells were collected from ischemic muscles d4 after ischemia and analyzed for F4/80 and Ly-6C expression. In accordance with observations described above, overall proportion of macrophages among CD45+ cells did not differ between *wt* and *tsp-1* ^−/−^ mice (respectively 86% and 84% of CD45+ cells). However, a transition from F4/80(lo)Ly-6C(hi) to F4/80(hi)Ly-6C(lo) macrophages was observed in *tsp-1* ^−/−^ mice when compared to *wt* (fig. 7A, fig. S4B and quantification in fig. 7B, n = 25). F4/80(lo)Ly-6C(hi) cells still consisted in an homogeneous SSC(lo)CD11b(hi) macrophage population at d4 under CD45+ isolation (fig. S4A), and transition in macrophage subsets in *tsp-1* ^−/−^ mice was confirmed under Ficoll isolation (fig. S4C, n = 5).

Inflammatory cytokine mRNA expression of intra-tissular F4/80(lo)Ly-6C(hi) and F4/80(hi) macrophage subsets at d4 were then compared using RT-qPCR, in both genotypes. As shown in fig. 7C, F4/80(hi) macrophages exhibited a reduced IL-1ß and TNF-α cytokine expression and an increased IL-10 and PPAR-γ expression in both genotypes, compared to F4/80(lo)Ly-6C(hi) macrophages. TGF-ß expression remained unchanged in both subsets in both genotypes. Transition in cytokine expression profile was associated with a diminished expression of inducible nitric oxide synthase and an increased expression of arginase 1 in F4/80(hi) macrophage subset. Expression levels of all these genes did not differ between both genotypes in F4/80(lo)Ly-6C(hi) subset (not shown). Consistently with an enrichment of Ly-6C(lo) macrophages among F4/80(hi) macrophages (fig. 7A), F4/80(hi) macrophages exhibited a reduced expression of pro-inflammatory cytokines in *tsp-1^−/−^* mice. Altogether, our data demonstrate a shift toward a reduced Ly-6C(hi) pro-inflammatory activation state in *tsp-1* ^−/−^ macrophages at d4, when compared to *wt*.

### Depletion of circulating monocytes reverses tissue protection in *tsp-1* ^−/−^ mice

**Figure 8:**
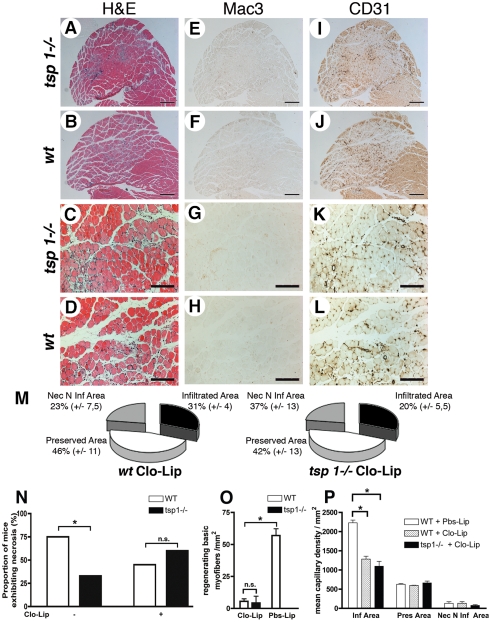
Depletion of circulating monocytes reverses tissue protection in tsp-1−/− mice. Histological analysis of gastrocnemius muscle sections at d4 after ischemia under Clo-Lip treatment. (A–D) show H&E staining. (E–H) and (I–L) show immunostainings of adjacent sections for macrophages using Mac-3 Ab, and endothelial cells using CD31 Ab, respectively. (A, B; E, F; I, J) scale bar = 500 µm; (C, D; G, H; K, L) scale bar = 200 µm. (M) Quantification of histologically different area types ratio; mean±S.E.M. are shown, n = 5. (N) Proportion of mice exhibiting macroscopic necrosis under Clo-Lip treatment, d4 after ischemia. * = p<0.05, n = 10. (O) Quantification of regenerating basic myofibers in necrotic areas, mean±S.E.M. are shown, * = p<0.05, n = 10. (P) Quantification of capillary density under Clo-Lip treatment in the different area types in gastrocnemius muscles, d4 after ischemia; Pbs-Lip injected mice served as control. Mean±SEM are shown. * = p<0.05; n = 5.

As macrophages in ischemic tissues exhibited distinct phenotypes in *wt* and *tsp-1* ^−/−^ animals, we next sought to evaluate their potential role in the tissue protection described in *tsp-1* ^−/−^ mice. We selectively depleted circulating monocytes in *tsp-1* ^−/−^ and *wt* mice using clodronate-containing liposomes (Clo-Lip) [35]. White blood cells counts on blood samples demonstrated that monocyte depletion was selective and equally efficient in both groups (Table S1). As expected, macrophage infiltrate almost disappeared (fig. 8F&8H) as well as phagocyted myofibers in ischemic muscles of *wt* mice (fig. 8B&8D). Regenerating basic myofibers were almost completely absent (fig. 8D&8O) and capillary density decreased (fig. 8L&8P). Taken together, these data highlight the crucial role played by macrophages in muscle healing from ischemia, both promoting tissue regeneration and angiogenesis.

Furthermore, we observed that monocyte depletion abolished clinical protection in *tsp-1* ^−/−^ mice. Indeed, macroscopic necrosis was no longer different in both genotypes under Clo-Lip treatment (fig. 8N). Histological analysis confirmed the complete loss of tissue protection in *tsp-1* ^−/−^ mice (fig. 8A&8B; see quantifications in fig. 8M). When assessing capillary density, no difference was observed between *wt* and *tsp-1* ^−/−^ in infiltrated, preserved and necrotic non-infiltrated areas (fig. 8I–8L&8P). Abolition of muscle regeneration was also similar in both genotypes (fig. 8O). In these experiments, it should be noted that *tsp-1* mRNA was still expressed at high levels, since its expression in endothelial cells and myofibers was not affected (not shown).

Altogether, these results demonstrate that macrophages are necessary during the post-ischemic healing process and that tissue protection observed in *tsp-1* ^−/−^ mice was critically dependent on distinct macrophage properties.

## Discussion

Physiopathology of critical limb ischemia (CLI) is complex, involving both impairment of angiogenesis and tissue regeneration, and a pro-inflammatory state with potential deleterious effects [2], [6], [7]. In humans, atrophic lower limb muscles show attenuated proangiogenic and regenerative signals and upregulate anabolic/survival pathways, which impairs angiogenesis and tissue regeneration [7]. However, some myofibers are nevertheless able to regenerate and produce high amounts of proangiogenic and prosurvival factors, such as VEGF [6], [7], therefore pointing out the rational for novel pharmacological approaches that would both prevent tissue damage and promote regeneration. Here we focused on TSP-1, which is highly expressed during CLI and shares the same expression pattern in endothelial cells and macrophages in both humans and mice. Using a genetic model, we report that *tsp-1* deficiency causes a shift of macrophages infiltrating ischemic tissues toward a less pro-inflammatory phenotype, responsible for protection from necrosis, improved postischemic angiogenesis and better tissue regeneration. This defines TSP-1 as a potential target for therapeutic immuno-modulation in humans during CLI.

In addition, this study proposes a mechanism that links macrophage activation state and tissue damage during critical leg ischemia. Macrophages play a crucial role during healing/regeneration processes [8]–[10], both phagocytosing debris from necrotic tissues and promoting tissue healing [6], [9], [30]. However macrophages constitute a heterogeneous population that differs in receptor expression, chemotactic properties and cytokine profile [34], [36]. In particular, Ly-6C(hi) monocytes/macrophages exhibit a pro-inflammatory cytokine profile and cytotoxic activities, whereas Ly-6C(lo) monocytes/macrophages show an anti-inflammatory profile and tissue repair activities [33], [34]. In our model of critical ischemia, a transition from Ly-6C(hi) to Ly-6C(lo) macrophages was observed, associated with a raise in F4/80 expression. This is in line with previous observations made during myocardial infarction and toxin-induced muscle injury [8], [9]. However we here describe beneficial effects of modulating of the Ly-6C(hi)/Ly-6C(lo) macrophage ratio during critical ischemia. We showed that *tsp-1* ^−/−^ mice displayed a significant shift of macrophage population infiltrating ischemic tissues toward the Ly-6C(lo) subset during CLI, exhibiting a diminished IL1-β and TNF-α pro-inflammatory cytokine mRNA expression, and an increased IL-10 and PPAR-γ mRNA expression when compared to Ly-6C(hi) macrophages. Also, iNOS mRNA expression was strongly reduced, whereas Arginase I mRNA was more expressed in Ly-6C(lo) macrophages. Remarkably, TGF-ß mRNA expression was shown to be unchanged between both subsets. We therefore believe that a transition from an activation state close to a classical activation state (M1 polarization) to an alternative activation state (M2 polarization) occurred in *tsp-1* ^−/−^ mice [34], [36]–[38]. We then showed that monocyte depletion abolished tissue protection in *tsp-1* ^−/−^ mice, thereby demonstrating that this protection from necrosis was mediated by macrophages. Deleterious effects of Ly-6C(hi) macrophages were previously shown in mice lacking the MCP-1/CCR2 pathway (responsible for the attraction of the Ly-6C(hi) subset in ischemic lesions) during myocardial, renal and cerebral ischemia [27], [39]–[41], in which protection was associated with a delayed macrophage infiltration and a reduced pro-inflammatory cytokine profile. Conversely, alternative macrophage activation state might be responsible for beneficial effects of PPAR-γ ligands in numerous models of ischemia [38], [42]–[44]. To our knowledge, this is the first time that beneficial effects of modulating the Ly-6C(hi)/Ly-6C(lo) macrophage ratio are described during critical ischemia. Whether tissue protection can be improved by further shifting the balance toward Ly-6C(lo) macrophages might be a major issue. Another key point might be to identify effectors of tissue necrosis and tissue protection produced by Ly-6C(hi) and Ly-6C(lo) macrophages, respectively. In particular M1 macrophage polarization is associated with an increased release of reactive oxygen species, higher expression of matrix metalloproteinases, and decreased VEGF expression when compared to M2 polarized macrophages [34], [37], [45].

Mechanisms that promote the transition from Ly-6C(hi) to Ly-6C(lo) macrophages during healing/regeneration process are subject to debate. We here show that macrophage density in ischemic lesions as well as the proportion of macrophages in the whole inflammatory cell population were similar between *tsp-1* ^−/−^ and *wt* mice. This does not support differential recruitment as the main mechanism responsible for modulating the Ly-6C(hi)/Ly-6C(lo) macrophage distribution. However, we observed a striking inhibition of necrotic myofiber phagocytosis in *tsp-1* ^−/−^ mice. This is to our knowledge the first time that a reduced phagocytotic ability of *tsp-1* ^−/−^ macrophages is described *in vivo*. This property was confirmed *in vitro*, reverted by recombinant TSP-1, and is consistent with previous results that emphasized the importance of TSP-1 during macrophage phagocytosis of necrotic cells [25]. However consequences of necrotic cells phagocytosis on macrophage cytokine expression profile are debated, depending on cell types and conditions used. Here we demonstrated that phagocytosis of necrotic muscle cell debris is a strong pro-inflammatory signal for macrophages. This is in line with previous studies which demonstrated that phagocytosis of whole necrotic cells (including organelles) induces a pro-inflammatory cytokine profile in macrophages, in contrast to necrotic cell membrane [8], [46]–[48]. As macrophages sequentially change their activation state in response to their microenvironment [36], our results indicate that TSP-1-dependent phagocytosis abilities participate in the intra-tissular modulation of macrophage activation state in this model of critical ischemia. Interestingly, both Ly-6C(hi) and Ly-6C(lo) subsets were sequentially recruited using different chemokine pathways in a mouse model of myocardial infarction [9], whereas an intra-tissular switch of macrophages mainly depending on phagocytosis was described in a mouse model of toxin-induced muscle necrosis [8]. Altogether, these data may indicate that mechanisms that control the Ly-6C(hi)/Ly-6C(lo) macrophage ratio are lesion and tissue dependent. In addition, TSP-1 also enhances expression of pro-inflammatory cytokines in macrophages [19] and regulates their migration *in vitro* [20], mechanisms that may also be partially involved in the modulation of macrophage activities in *tsp-1* ^−/−^ mice.

Macrophages are known to be involved in many angiogenic processes [31]. During non critical (without necrosis) hind limb ischemia, macrophages have a crucial role in mediating arteriogenesis in tight muscles [49]. In the present study we described macrophages to be highly present in postischemic infiltrates in calf muscles during critical ischemia. Capillary density was strongly improved in infiltrated areas, whereas macrophage depletion was responsible for both postischemic angiogenesis and muscle regeneration impairment. This is in accordance with previous studies in mice lacking the CCR2/MCP-1 pathway [28], [30] and studies of monocyte depletion during other types of muscle injuries [10], and emphasizes the crucial role of macrophages in postischemic angiogenesis and muscle regeneration during CLI. Interestingly TSP-1 displays strong anti-angiogenic properties in cancer [12], where macrophages are highly present [31]. A recent study also demonstrated an increased NO-mediated postischemic vasorelaxation in *tsp-1* ^−/−^ mice, responsible for tissue protection in a model of ischemic skin flaps [16]. However *tsp-1* ^−/−^ mice were still protected from ischemia under L-NAME treatment when compared to *wt*, showing that additional mechanisms were involved. In our study macrophage depletion fully reversed *tsp-1* ^−/−^ phenotype after ischemia, thereby demonstrating that anti-angiogenic properties of TSP-1 were mediated by macrophages.

Interestingly, we here show that *tsp-1* ^−/−^ mice exhibited a shift toward less pro-inflammatory postischemic infiltrates, whereas *in vitro* and *in vivo* studies have linked TSP-1 with the resolution of the inflammatory process [18], [50]–[52]. Frangogiannis *et al.* demonstrated a prolonged postischemic macrophage infiltrate in *tsp-1* ^−/−^ mice during myocardial infarction [17] and Lamy *et al.* demonstrated a prolonged inflammatory phase in *tsp-1* ^−/−^ mice in a model of oxazolone-induced skin inflammation [18]. Postischemic infiltrates mostly contain macrophages and necrotic myofibers at d4, and very few CD3+ cells (data not shown). Conversely oxazolone-induced skin infiltrates and post-ischemic tissues at later stages contain a high proportion of lymphocytes [18]. This could explain a differential role for TSP-1 in modulating these different types of infiltrates: TSP-1-dependent phagocytosis of necrotic lymphocytes induces an anti-inflammatory response in macrophages [25], whereas phagocytosis of necrotic muscle cells promotes a pro-inflammatory response.

In conclusion we describe here TSP-1 as a new relevant physiological target during critical leg ischemia in humans. Furthermore, we describe the modulation of postischemic macrophage activation state as a new potential therapeutic approach to protect tissues from necrosis and promote tissue repair during critical ischemia. Finally our data also suggest that phagocytosis of necrotic muscle debris plays a crucial role in regulating the intra-tissular macrophage activation state during critical leg ischemia.

## Methods

### Animals

*Thrombospondin-1*−/− mice were on a C57/Bl6 background as described previously [53]. All experiments were performed on 12 to 18 weeks old males.

This study conforms to the standards of INSERM (the French National Institute of Health) regarding the care and use of laboratory animals, was performed in accordance with European Union Council Directives (86/609/EEC) and was approved by the institutional research ethics committee IDF - Paris - Comité 1 **(**ref : 2008004**)**.

### Hind limb ischemia procedures

Unilateral critical ischemia was generated by ligation/excision of the femoral artery as previously described [54]. Mice were anesthetized with i.p. injection of ketamin 2 mg (Imalgene) and xylazine 0.2 mg (Rompun). After skin incision, the superficial epigastric artery was ligated (Ethicon 6-0, Vicryl). The proximal end of the left femoral artery and the distal portion of the saphenous artery were ligated. The femoral artery was then excised. Femoral vein and nerve were not preserved during surgical procedures. After surgery, the skin was closed with interrupted 6.0 proline sutures.

### Macroscopic necrosis

The incidence of hind limb macroscopic necrosis was determined at d2, d7, d14 and d21 after femoral artery excision (*n* = 19 mice per group).

### Histological analyses and immunohistochemistry

After sacrifice at d4, gastrocnemius muscles were fixed, dehydrated and paraffin-embedded. Serial adjacent 7 µm cross sections were generated through the midportion of the muscle for H&E staining and Mac-3 immunostaining (that labels macrophages). Myofibers with pale cytoplasm and loss of peripheral nuclei were defined as necrotic as previously described [30]. Macrophages were stained using a rat anti-mouse Mac-3 monoclonal Ab (BD Biosciences, dilution 1/75) and revealed with a secondary biotin-conjugated goat anti-rat Ab (Jackson Immunoresearch, dilution 1/200). ABC-peroxydase complex (Vector Laboratories) was used for signal amplification.

Three types of area were observed (fig. S2A): an infiltrated area (that display necrosis and macrophage infiltrate) (fig. S2B&2C), a preserved area (normal histology) (fig. S2D&2E) and a necrotic non-infiltrated area (that display necrosis without macrophage infiltrate) (fig. S2F&2G). Images of each area were digitally captured on H&E slides using a Leica DM 4000B microscope equipped with a DFC 420 camera and the Application Suite 2.7.1 software. The surface of each area type was quantified in each mouse using Metamorph software and reported as percentage of the entire section surface (*n* = 9) (see fig. S2A). Regenerating basic myofibers were quantified on whole muscle section as previously described [8] for each mouse (*n* = 9). For macrophage density, macrophages were counted on 5 digitally captured non-overlapping ×20 magnification fields in the infiltrated area, and reported as a single value/mm^2^ in each animal (*n* = 9). Necrotic myofibers containing two or more macrophages were defined as phagocyted and expressed as a percentage of total necrotic myofibers, on digitally captured images of non-overlapping fields (×20 magnification, 4 fields per animal, *n* = 9).

Assessment of capillary density. Biotin-conjugated rat anti-mouse CD31 Ab (BD Biosciences, dilution 1∶50) and Cy3-conjugated mouse monoclonal anti α-smooth muscle actin Ab (Sigma-Aldrich, dilution 1/100) were used to identify endothelial cells and vascular smooth muscle cells, respectively. For capillary density quantification, nonoverlapping ×20 fields (5 per area) were digitally captured. Capillaries were counted using the software IP lab 3.2.4.

**Western Blot analyses** of TSP-1 expression were performed as previously described [26] excepted that attophos substrate (Promega) was used. The specificity of antibody binding was verified in *tsp-1* ^−/−^ mice.

***In situ*** **hybridization** procedures were performed as previously described [55]. A 1186 bp cDNA fragment (nucleotide 151-1336) was used to generate a ^35^S-RNA antisense mouse *tsp-1* probe. A sense probe was used as a negative control.

### Microangiography

Mice were anesthetized (40 µl i.p. sodium pentobarbital) and longitudinal laparotomy was performed to introduce a polyethylene catheter into the abdominal aorta to inject contrast medium (barium sulfate, 1 g/mL). Angiography of hind limbs was then performed and images (two per animal) were acquired using a high-definition digital X-ray transducer. Computerized quantification of vessel density was then performed and expressed as a ratio from ischemic to non ischemic leg of the percentage of pixels per image occupied by vessels in the quantification area.

### Bone marrow-derived macrophage isolation and differentiation

Primary culture of murine bone marrow macrophages were harvested from femur of 12 to 18 week-old *tsp-1* ^−/−^ and *wt* mice as described previously [36]. Briefly, the marrow cells were flushed from the bone with a 26G needle connected to a syringe filled with RPMI 1640 supplemented with 1% antibiotic mixture (penicillin, streptomycin, neomycin). Following centrifugation over Ficoll, the cells were cultured in DMEM medium (Gibco 31885) supplemented with 1% antibiotic mixture, 10% FBS and 20% of L-929-conditioned medium (source of M-CSF-1). Non-adherent cells were collected after 24 h, seeded on coverslips, and differentiated for 7 days in polystyrene culture plates, changing the medium once on d4. The resulting cell population was >95% F4/80 and 98% Mac-3 positive as assessed by flow cytometry.

### Phagocytosis

After overnight priming with IFN-γ (100 U/ml), bone marrow macrophages cells were washed and activated overnight with 100 ng/ml LPS. Fluorescent latex beads (Sigma L3030, 2 µm) were seeded on macrophages (three beads per one macrophage) for 3 h at 37°C with or without 2,5 µg/ml recombinant TSP-1 (US Biological). macrophage cultures were washed four times to remove noningested material and fixed using 4% paraformaldehyde. Coverslips were mounted with Moviol/ToPro (Invitrogen) and analyzed in sequential scanning mode using a ×40 objective lens with a Leica TCS SP2 confocal microscope equipped with three external lasers (488, 543 and 633 nm) (Leica Microsystems). The number of Latex-containing cells was expressed as the percentage of total cells. Five nonoverlapping fields were averaged and reported as a single value for each well.

For inflammation assays, necrotic myogenic precursor cells (lyzed by three cycles of freeze-thawing) were seeded on IFN-γ-activated macrophages (ratio 3∶1) for 3 h at 37°C. macrophage cultures were washed three times to remove noningested material and further cultured in serum-free medium (GIBCO 31885) for 24 h prior to collection of conditioned media. TNF-α, IL-6 and IL-10 were quantified in the supernatant by enzyme-linked immunoabsorbent assay (Quantikine, R&D systems). To inhibit phagocytosis, macrophages were pre-treated with 10 µg/ml colchicine (Sigma-Aldrich) for 30 min, as previously described [32].

**Isolation of monocytes/macrophages from muscle and RNA preparation** were performed as previously described [8]. Briefly, muscles were dissociated in DMEM containing collagenase B 0.2% (Roche Diagnostics Gmbh) at 37°C for 90 min, filtered and counted. Inflammatory cells cells were isolated using CD45+ magnetic sorting (Milteny Biotec) or centrifugation over Ficoll and stained with the following combinations of antibodies : {PE-conjugated F4/80 (AbD Serotec) and FITC-conjugated Ly-6G/ Ly-6C (only Ly-6C is expressed by macrophages [8]) (eBioscience)} antibodies, or {APC-conjugated F4/80 (eBioscience), PE-Cy7-conjugated CD45 (eBioscience), FITC-conjugated Ly-6G/ Ly-6C (eBioscience) and PE-conjugated CD11b (BD Pharmingen)} antibodies, in association with a CD16/CD32 Fc block antibody (BD Pharmigen). Analysis was performed using a cytometer (MoFlo and Cyan, Dako-Cytomation). Using either CD45+ magnetic sorting or Ficoll isolation, F4/80(lo) Ly-6C(hi) cells consisted at d4 in an homogeneous monocyte/macrophage population, as indicated by their mononuclearity read as low orthogonal (side) scatter in the flow cytometer and their myeloid nature, as indicated by high level of CD11b expression [33] (fig. S3B & fig. S4A).

### RT-qPCR

0.5 µg of total RNA was reverse transcribed using Superscript II reverse transcriptase. Each cDNA preparation was amplified using a iQ™ SYBR green supermix (Bio-Rad) and the following specific primers (sense and antisense, respectively): β2 microglobulin, 5′-CAGTTCCACCCGCCTCAC-3′ and 5′-CACATGTCTCGATCCCAG-3′; TNF-α, 5′-AAAGATGGGGGGCTTCCAGAACTC-3′ and 5′-TGAGATAGCAAATCGGCTGACGG-3′; IL-1β, 5′-TGACGTTCCCATTAGACAACTG-3′ and 5′-CCGTCTTTCATTACACAGGACA-3′; IL-10, 5′-ACCAGCTGGACAACATACTGC-3′ and 5′-TCACTCTTCACCTGCTCCACT-3′; PPAR-γ, 5′-AGGCCGAGAAGGAGAAGCTGTTG -3′ and 5′-TGGCCACCTCTTTGCTCTGCTC-3′, TFG-ß1, 5′-TGCGCTTGCAGAGATTAAAA-3′ and 5′-CGTCAAAAGACAGCCACTCA-3′; iNOS, 5′-GAAGAAAACCCCTTGTGCTG-3′ and 5′-TCCAGGGATTCTGGAACATT-3′; Arg I, 5′-CAGAAGAATGGAAGAGTCAG-3′ and 5′-CAGATATGCAGGGAGTCACC-3′. PCR conditions were: 95°C for 15 min and then 95°C for 15 s, 60°C for 30 s and 72°C for 45 s repeated for 40 cycles. Melting curves were obtained. Amplification was performed using a iCycler equipped with a MyiQ™ optical module (Bio-Rad). Analysis of the relative expression of each target gene was related to β2 microglobulin expression using the iQ™5 Optical system software (Bio-Rad).

**Macrophage depletion** was achieved using clodronate (Cl2-MDP)-containing liposomes as previously described [35], [56]. This method allows the specific depletion in monocytes and macrophages, which undergo apoptosis upon phagocytosis of Cl2MDP liposomes. Clo-Lip were injected i.v. 12 h before femoral artery ligation in *tsp-1* ^−/−^ and *wt* mice (250 µl, 10 mice per group). Considering the half-life of this product (48 h), injection was repeated at d2 after ligation and mice were sacrificed at d4 for clinical, histological and immunohistochemical analyses. PBS-containing liposomes (Pbs-Lip) were used as a negative control in *wt* mice (10 mice). Clodronate was a gift of Roche Diagnostics GmbH.

### White blood cell counts

Blood samples were taken from *tsp-1* ^−/−^ and *wt* mice prior to Clo-Lip injection and at d4 after femoral artery ligation (*n* = 10 per group). After May Grünwald Giemsa staining, percentage of monocyte, lymphocyte and neutrophil were quantified by a hematologist. At least 200 cells were counted for each sample.

### Statistical analyses

All experiments were conducted in a blinded-manner for both genotype and groups of mice, and performed using cultures or animals in at least three independent experiments. Staview (SAS institute Inc.) and Prism 4.0 (GaphPad software, Inc) were used for statistical analyses. Continuous variables are reported as Mean±SEM. Incidence of macroscopic necrosis was estimated by the Kaplan-Meier method, and differences were assessed by means of the log-rank test. Wilcoxon test was used to compare proportions of macrophage subsets in both genotypes (fig. 7), and Mann-Whitney was used to compare *tsp-1* ^−/−^ from *wt* mice in other experiments. Statistical significance was set at *p*<0.05.

## Supporting Information

Figure S1 Description of the healing/regeneration process in gastrocnemius muscle in response to ischemia. Histological analyses of gastrocnemius muscles sections at d4, d6, d16 and d21 after ischemia. (A, D, G, J) show H&E staining. (B, E, H, K) and (C, F, I, L) show immunostainings of adjacent sections for macrophages using a Mac-3 Ab, and endothelial cells using a CD31 Ab, respectively. Scale bar = 200 µm.
(8.83 MB TIF)

Figure S2 Three different types of area are observed in gastrocnemius muscle at d4 after ischemia. (A) Cross sections were generated through the midportion of gastrocnemius d4 after ischemia and stained with H&E. (A) representative section of the right part of wt mice gastrocnemius is shown (scale bar = 500 µm). Adjacent sections were immunostained for macrophages using Mac-3 Ab. (B–G) Higher magnification of infiltrated area (necrotic myofibers+macrophage infiltrate) (B, C), preserved area (normal histology) (D, E) and necrotic non-infiltrated area (necrotic myofibers+absence of macrophage infiltrate) (F, G), either stained with H&E (B, D, F) or immunostained for Mac-3 (C, E, G); scale bar = 100 µm. Quantification. Surfaces of infiltrated area (black stroke) and preserved area (green stroke) were quantified and reported as percentage of the entire section surface in each mouse. The remainder of surface percentage was attributed to necrotic non-infiltrated area ( = 100%- infiltrated area (%)- preserved area (%) in each mouse).
(14.85 MB TIF)

Figure S3 Kinetic analysis of intra-tissular macrophage activation state during ischemia. (A) Mononuclear cells were isolated from ischemic muscles of C57Bl6 mice (Charles-River) using centrifugation over Ficoll, and analyzed by FACS for F4/80 and Ly-6C expression (n = 3 per time point). (B) SSC/FSC characteristics and CD11b expression of Ficoll-isolated F4/80(lo) Ly-6C(hi) cells at d4, showing an homogeneous SSC(lo) CD11b(hi) macrophage population.
(4.71 MB TIF)

Figure S4 Thrombospondin-1−/− mice exhibit a less pro-inflammatory macrophage activation state in response to ischemia. Additional experiments. (A) SSC/FSC characteristics and CD11b expression of CD45+ F4/80(lo) Ly-6C(hi) cells isolated from ischemic muscles at d4, in both genotypes, showing an homogeneous SSC(lo)CD11b(hi) macrophage population. (B) FACS analyses of CD45+ cells isolated from ischemic muscles at d4, stained for F4/80 and Ly-6C expression. Additional examples of two independent experiments are shown (n = 5 mice). (C) Upper panel, representative FACS analyses of Ficoll-isolated mononuclear cells from ischemic muscles of one mouse from both genotypes at d4, stained for F4/80 and Ly-6C. Lower panel, quantification of F4/80(lo)Ly-6C(hi), F4/80(hi)Ly-6C(hi) and F4/80(hi)Ly-6C(lo) macrophage proportions in both genotypes (n = 5).
(6.79 MB TIF)

Table S1 Clo-Lip induced monocyte depletion is identical in both genotypes. Percentage of monocytes, neutrophils and lymphocytes in total white blood cells, counted on blood samples in both genotypes, at baseline (before injection) and d4 after ischemia under Clo-Lip treatment. Mean±SEM are reported. ** = p<0.01 Clo-Lip vs. baseline; n = 10.
(0.02 MB XLS)

## References

[1] Dormandy JA, Rutherford RB (2000). Management of peripheral arterial disease (PAD). TASC Working Group. TransAtlantic Inter-Society Consensus (TASC).. J Vasc Surg.

[2] Barani J, Nilsson JA, Mattiasson I, Lindblad B, Gottsater A (2005). Inflammatory mediators are associated with 1-year mortality in critical limb ischemia.. J Vasc Surg.

[3] Baumgartner I, Pieczek A, Manor O, Blair R, Kearney M (1998). Constitutive expression of phVEGF165 after intramuscular gene transfer promotes collateral vessel development in patients with critical limb ischemia.. Circulation.

[4] Lederman RJ, Mendelsohn FO, Anderson RD, Saucedo JF, Tenaglia AN (2002). Therapeutic angiogenesis with recombinant fibroblast growth factor-2 for intermittent claudication (the TRAFFIC study): a randomised trial.. Lancet.

[5] Rajagopalan S, Mohler E, Lederman RJ, Saucedo J, Mendelsohn FO (2003). Regional Angiogenesis with Vascular Endothelial Growth Factor (VEGF) in peripheral arterial disease: Design of the RAVE trial.. Am Heart J.

[6] Rissanen TT, Vajanto I, Hiltunen MO, Rutanen J, Kettunen MI (2002). Expression of vascular endothelial growth factor and vascular endothelial growth factor receptor-2 (KDR/Flk-1) in ischemic skeletal muscle and its regeneration.. Am J Pathol.

[7] Tuomisto TT, Rissanen TT, Vajanto I, Korkeela A, Rutanen J (2004). HIF-VEGF-VEGFR-2, TNF-alpha and IGF pathways are upregulated in critical human skeletal muscle ischemia as studied with DNA array.. Atherosclerosis.

[8] Arnold L, Henry A, Poron F, Baba-Amer Y, van Rooijen N (2007). Inflammatory monocytes recruited after skeletal muscle injury switch into antiinflammatory macrophages to support myogenesis.. J Exp Med.

[9] Nahrendorf M, Swirski FK, Aikawa E, Stangenberg L, Wurdinger T (2007). The healing myocardium sequentially mobilizes two monocyte subsets with divergent and complementary functions.. J Exp Med.

[10] Summan M, Warren GL, Mercer RR, Chapman R, Hulderman T (2006). Macrophages and skeletal muscle regeneration: a clodronate-containing liposome depletion study.. Am J Physiol Regul Integr Comp Physiol.

[11] Engstrom G, Site-Flondell D, Lindblad B, Janzon L, Lindgarde F (2004). Risk of treatment of peripheral arterial disease is related to inflammation-sensitive plasma proteins: a prospective cohort study.. J Vasc Surg.

[12] Esemuede N, Lee T, Pierre-Paul D, Sumpio BE, Gahtan V (2004). The role of thrombospondin-1 in human disease.. J Surg Res.

[13] Agah A, Kyriakides TR, Lawler J, Bornstein P (2002). The lack of thrombospondin-1 (TSP1) dictates the course of wound healing in double-TSP1/TSP2-null mice.. Am J Pathol.

[14] Lawler J (2002). Thrombospondin-1 as an endogenous inhibitor of angiogenesis and tumor growth.. J Cell Mol Med.

[15] Lawler J, Detmar M (2004). Tumor progression: the effects of thrombospondin-1 and -2.. Int J Biochem Cell Biol.

[16] Isenberg JS, Hyodo F, Matsumoto K, Romeo MJ, Abu-Asab M (2007). Thrombospondin-1 limits ischemic tissue survival by inhibiting nitric oxide-mediated vascular smooth muscle relaxation.. Blood.

[17] Frangogiannis NG, Ren G, Dewald O, Zymek P, Haudek S (2005). Critical role of endogenous thrombospondin-1 in preventing expansion of healing myocardial infarcts.. Circulation.

[18] Lamy L, Foussat A, Brown EJ, Bornstein P, Ticchioni M (2007). Interactions between CD47 and thrombospondin reduce inflammation.. J Immunol.

[19] Yamauchi Y, Kuroki M, Imakiire T, Abe H, Uchida H (2002). Thrombospondin-1 differentially regulates release of IL-6 and IL-10 by human monocytic cell line U937.. Biochem Biophys Res Commun.

[20] Mansfield PJ, Suchard SJ (1994). Thrombospondin promotes chemotaxis and haptotaxis of human peripheral blood monocytes.. J Immunol.

[21] Moodley Y, Rigby P, Bundell C, Bunt S, Hayashi H (2003). Macrophage recognition and phagocytosis of apoptotic fibroblasts is critically dependent on fibroblast-derived thrombospondin 1 and CD36.. Am J Pathol.

[22] Ren Y, Savill J (1995). Proinflammatory cytokines potentiate thrombospondin-mediated phagocytosis of neutrophils undergoing apoptosis.. J Immunol.

[23] Stern M, Savill J, Haslett C (1996). Human monocyte-derived macrophage phagocytosis of senescent eosinophils undergoing apoptosis. Mediation by alpha v beta 3/CD36/thrombospondin recognition mechanism and lack of phlogistic response.. Am J Pathol.

[24] Ren Y, Stuart L, Lindberg FP, Rosenkranz AR, Chen Y (2001). Nonphlogistic clearance of late apoptotic neutrophils by macrophages: efficient phagocytosis independent of beta 2 integrins.. J Immunol.

[25] Bottcher A, Gaipl US, Furnrohr BG, Herrmann M, Girkontaite I (2006). Involvement of phosphatidylserine, alphavbeta3, CD14, CD36, and complement C1q in the phagocytosis of primary necrotic lymphocytes by macrophages.. Arthritis Rheum.

[26] Favier J, Germain S, Emmerich J, Corvol P, Gasc JM (2005). Critical overexpression of thrombospondin 1 in chronic leg ischaemia.. J Pathol.

[27] Dimitrijevic OB, Stamatovic SM, Keep RF, Andjelkovic AV (2007). Absence of the chemokine receptor CCR2 protects against cerebral ischemia/reperfusion injury in mice.. Stroke.

[28] Contreras-Shannon V, Ochoa O, Reyes-Reyna SM, Sun D, Michalek JE (2007). Fat accumulation with altered inflammation and regeneration in skeletal muscle of CCR2−/− mice following ischemic injury.. Am J Physiol Cell Physiol.

[29] Ochoa O, Sun D, Reyes-Reyna SM, Waite LL, Michalek JE (2007). Delayed angiogenesis and VEGF production in CCR2−/− mice during impaired skeletal muscle regeneration.. Am J Physiol Regul Integr Comp Physiol.

[30] Shireman PK, Contreras-Shannon V, Ochoa O, Karia BP, Michalek JE (2007). MCP-1 deficiency causes altered inflammation with impaired skeletal muscle regeneration.. J Leukoc Biol.

[31] Dirkx AE, Oude Egbrink MG, Wagstaff J, Griffioen AW (2006). Monocyte/macrophage infiltration in tumors: modulators of angiogenesis.. J Leukoc Biol.

[32] Lucas M, Stuart LM, Zhang A, Hodivala-Dilke K, Febbraio M (2006). Requirements for apoptotic cell contact in regulation of macrophage responses.. J Immunol.

[33] Sunderkotter C, Nikolic T, Dillon MJ, Van Rooijen N, Stehling M (2004). Subpopulations of mouse blood monocytes differ in maturation stage and inflammatory response.. J Immunol.

[34] Gordon S, Taylor PR (2005). Monocyte and macrophage heterogeneity.. Nat Rev Immunol.

[35] van Amerongen MJ, Harmsen MC, van Rooijen N, Petersen AH, van Luyn MJ (2007). Macrophage depletion impairs wound healing and increases left ventricular remodeling after myocardial injury in mice.. Am J Pathol.

[36] Stout RD, Jiang C, Matta B, Tietzel I, Watkins SK (2005). Macrophages sequentially change their functional phenotype in response to changes in microenvironmental influences.. J Immunol.

[37] Gordon S (2003). Alternative activation of macrophages.. Nat Rev Immunol.

[38] Odegaard JI, Ricardo-Gonzalez RR, Goforth MH, Morel CR, Subramanian V (2007). Macrophage-specific PPARgamma controls alternative activation and improves insulin resistance.. Nature.

[39] Furuichi K, Wada T, Iwata Y, Kitagawa K, Kobayashi K (2003). CCR2 signaling contributes to ischemia-reperfusion injury in kidney.. J Am Soc Nephrol.

[40] Dewald O, Zymek P, Winkelmann K, Koerting A, Ren G (2005). CCL2/Monocyte Chemoattractant Protein-1 regulates inflammatory responses critical to healing myocardial infarcts.. Circ Res.

[41] Hayasaki T, Kaikita K, Okuma T, Yamamoto E, Kuziel WA (2006). CC chemokine receptor-2 deficiency attenuates oxidative stress and infarct size caused by myocardial ischemia-reperfusion in mice.. Circ J.

[42] Michalik L, Wahli W (2006). Involvement of PPAR nuclear receptors in tissue injury and wound repair.. J Clin Invest.

[43] Ricote M, Li AC, Willson TM, Kelly CJ, Glass CK (1998). The peroxisome proliferator-activated receptor-gamma is a negative regulator of macrophage activation.. Nature.

[44] Alleva DG, Johnson EB, Lio FM, Boehme SA, Conlon PJ (2002). Regulation of murine macrophage proinflammatory and anti-inflammatory cytokines by ligands for peroxisome proliferator-activated receptor-gamma: counter-regulatory activity by IFN-gamma.. J Leukoc Biol.

[45] Chan G, Bivins-Smith ER, Smith MS, Smith PM, Yurochko AD (2008). Transcriptome analysis reveals human cytomegalovirus reprograms monocyte differentiation toward an M1 macrophage.. J Immunol.

[46] Brouckaert G, Kalai M, Krysko DV, Saelens X, Vercammen D (2004). Phagocytosis of necrotic cells by macrophages is phosphatidylserine dependent and does not induce inflammatory cytokine production.. Mol Biol Cell.

[47] Scaffidi P, Misteli T, Bianchi ME (2002). Release of chromatin protein HMGB1 by necrotic cells triggers inflammation.. Nature.

[48] Fadok VA, Bratton DL, Guthrie L, Henson PM (2001). Differential effects of apoptotic versus lysed cells on macrophage production of cytokines: role of proteases.. J Immunol.

[49] Heil M, Eitenmuller I, Schmitz-Rixen T, Schaper W (2006). Arteriogenesis versus angiogenesis: similarities and differences.. J Cell Mol Med.

[50] Demeure CE, Tanaka H, Mateo V, Rubio M, Delespesse G (2000). CD47 engagement inhibits cytokine production and maturation of human dendritic cells.. J Immunol.

[51] Grimbert P, Bouguermouh S, Baba N, Nakajima T, Allakhverdi Z (2006). Thrombospondin/CD47 interaction: a pathway to generate regulatory T cells from human CD4+ CD25− T cells in response to inflammation.. J Immunol.

[52] Doyen V, Rubio M, Braun D, Nakajima T, Abe J (2003). Thrombospondin 1 is an autocrine negative regulator of human dendritic cell activation.. J Exp Med.

[53] Lawler J, Sunday M, Thibert V, Duquette M, George EL (1998). Thrombospondin-1 is required for normal murine pulmonary homeostasis and its absence causes pneumonia.. J Clin Invest.

[54] Couffinhal T, Silver M, Zheng LP, Kearney M, Witzenbichler B (1998). Mouse model of angiogenesis.. Am J Pathol.

[55] Le Jan S, Amy C, Cazes A, Monnot C, Lamande N (2003). Angiopoietin-like 4 is a proangiogenic factor produced during ischemia and in conventional renal cell carcinoma.. Am J Pathol.

[56] Van Rooijen N, Sanders A (1994). Liposome mediated depletion of macrophages: mechanism of action, preparation of liposomes and applications.. J Immunol Methods.

