# Supplementary figures and images for: Modulation of Macrophage Activation State Protects Tissue from Necrosis during Critical Limb Ischemia in Thrombospondin-1-Deficient Mice
Source: PLoS One. 2008 Dec 16;3(12):e3950. doi: 10.1371/journal.pone.0003950 (PMC2597179; doi:10.1371/journal.pone.0003950)

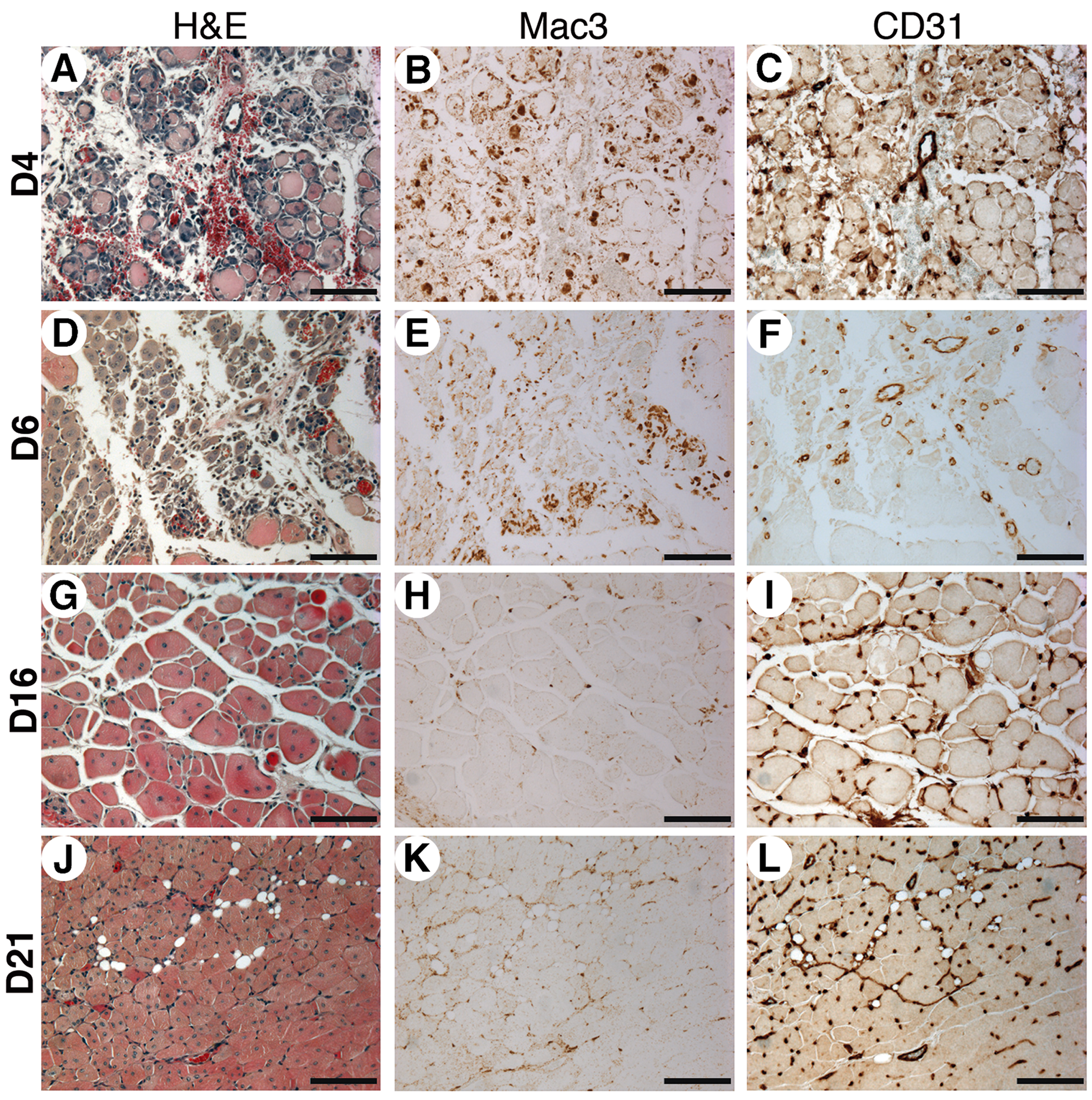

Supplement: Figure S1 — Description of the healing/regeneration process in gastrocnemius muscle in response to ischemia. Histological analyses of gastrocnemius muscles sections at d4, d6, d16 and d21 after ischemia. (A, D, G, J) show H&E staining. (B, E, H, K) and (C, F, I, L) show immunostainings of adjacent sections for macrophages using a Mac-3 Ab, and endothelial cells using a CD31 Ab, respectively. Scale bar = 200 µm. (8.83 MB TIF) [file pone.0003950.s001.tif]

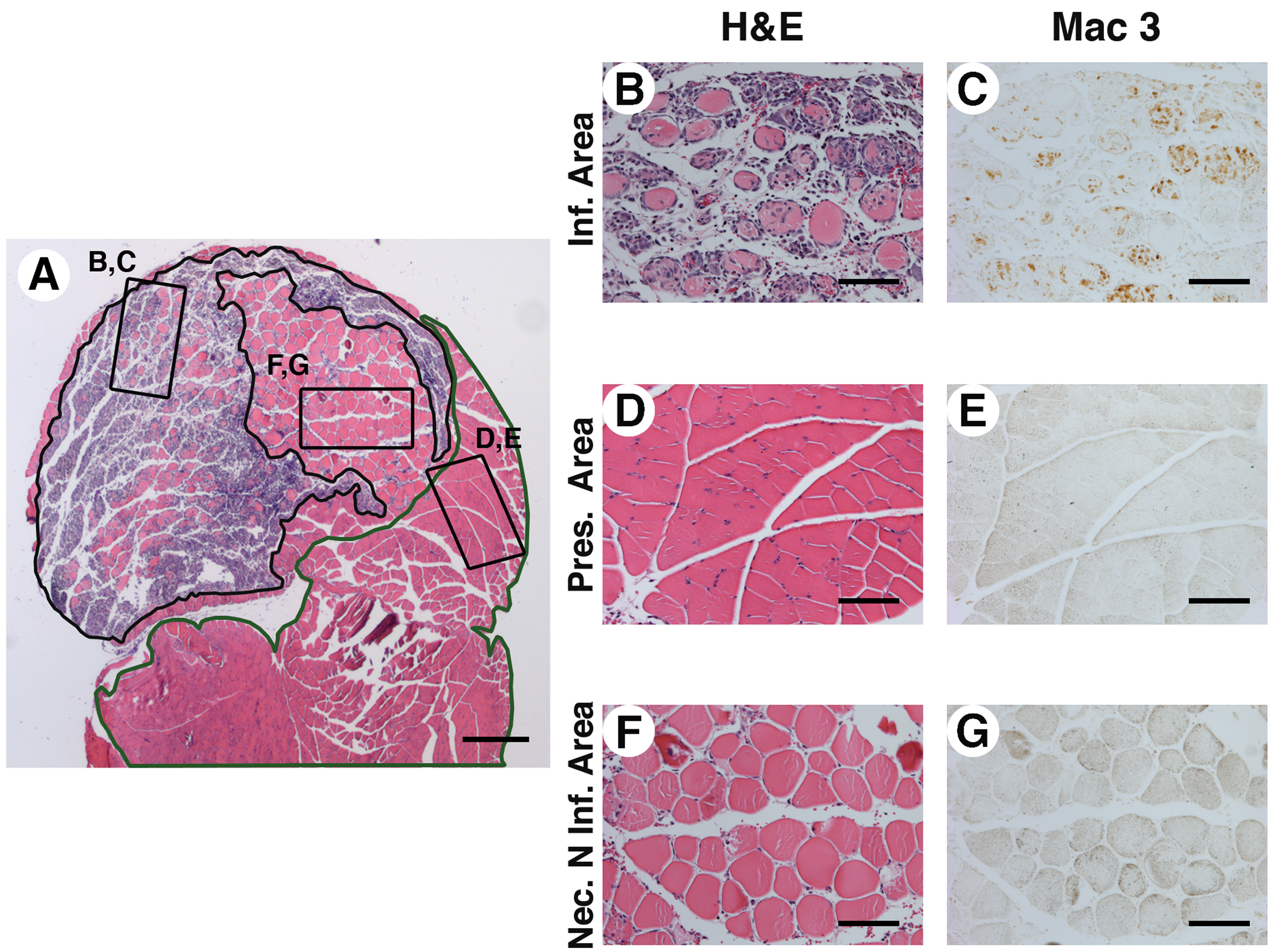

Supplement: Figure S2 — Three different types of area are observed in gastrocnemius muscle at d4 after ischemia. (A) Cross sections were generated through the midportion of gastrocnemius d4 after ischemia and stained with H&E. (A) representative section of the right part of wt mice gastrocnemius is shown (scale bar = 500 µm). Adjacent sections were immunostained for macrophages using Mac-3 Ab. (B–G) Higher magnification of infiltrated area (necrotic myofibers+macrophage infiltrate) (B, C), preserved area (normal histology) (D, E) and necrotic non-infiltrated area (necrotic myofibers+absence of macrophage infiltrate) (F, G), either stained with H&E (B, D, F) or immunostained for Mac-3 (C, E, G); scale bar = 100 µm. Quantification. Surfaces of infiltrated area (black stroke) and preserved area (green stroke) were quantified and reported as percentage of the entire section surface in each mouse. The remainder of surface percentage was attributed to necrotic non-infiltrated area ( = 100%- infiltrated area (%)- preserved area (%) in each mouse). (14.85 MB TIF) [file pone.0003950.s002.tif]

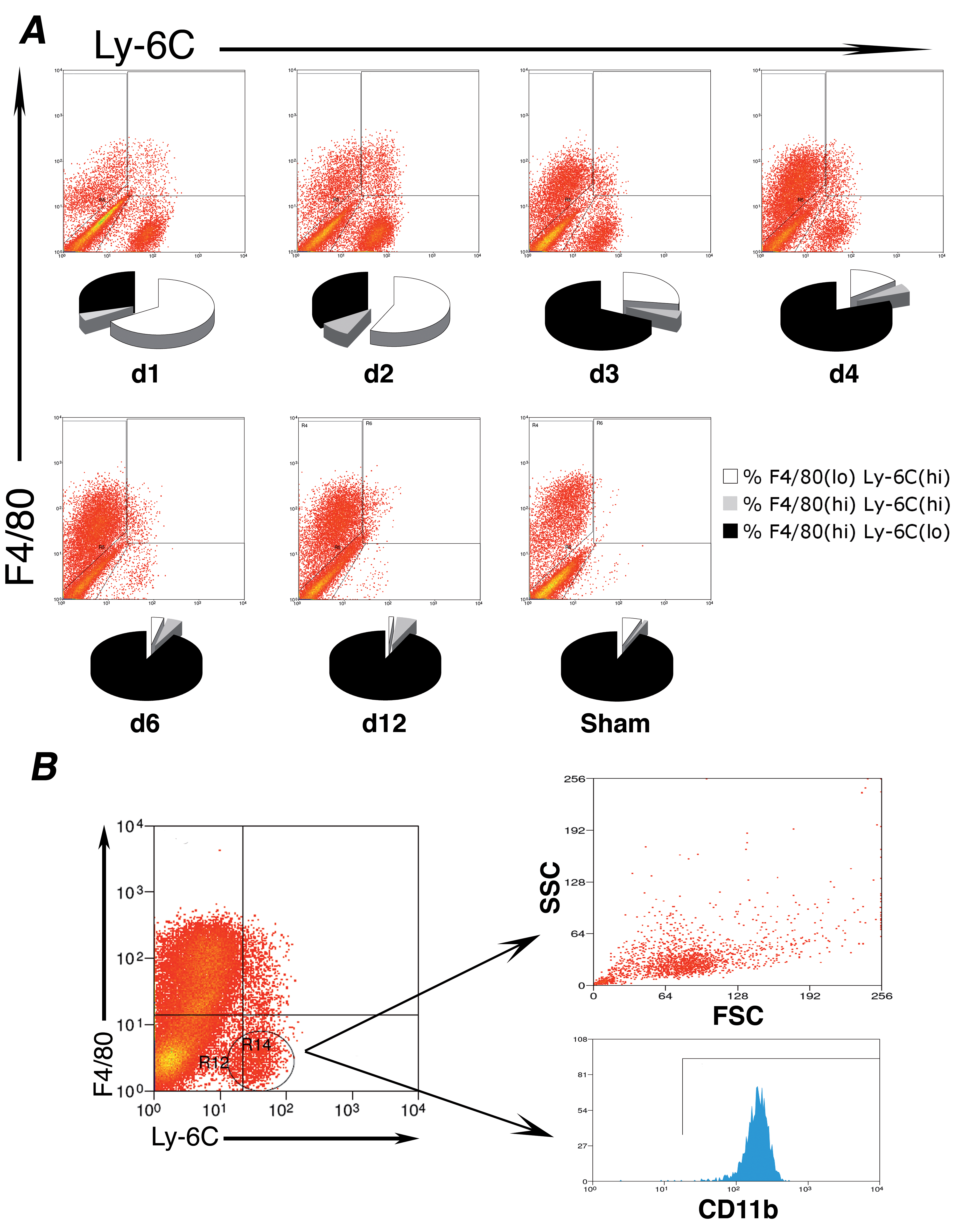

Supplement: Figure S3 — Kinetic analysis of intra-tissular macrophage activation state during ischemia. (A) Mononuclear cells were isolated from ischemic muscles of C57Bl6 mice (Charles-River) using centrifugation over Ficoll, and analyzed by FACS for F4/80 and Ly-6C expression (n = 3 per time point). (B) SSC/FSC characteristics and CD11b expression of Ficoll-isolated F4/80(lo) Ly-6C(hi) cells at d4, showing an homogeneous SSC(lo) CD11b(hi) macrophage population. (4.71 MB TIF) [file pone.0003950.s003.tif]

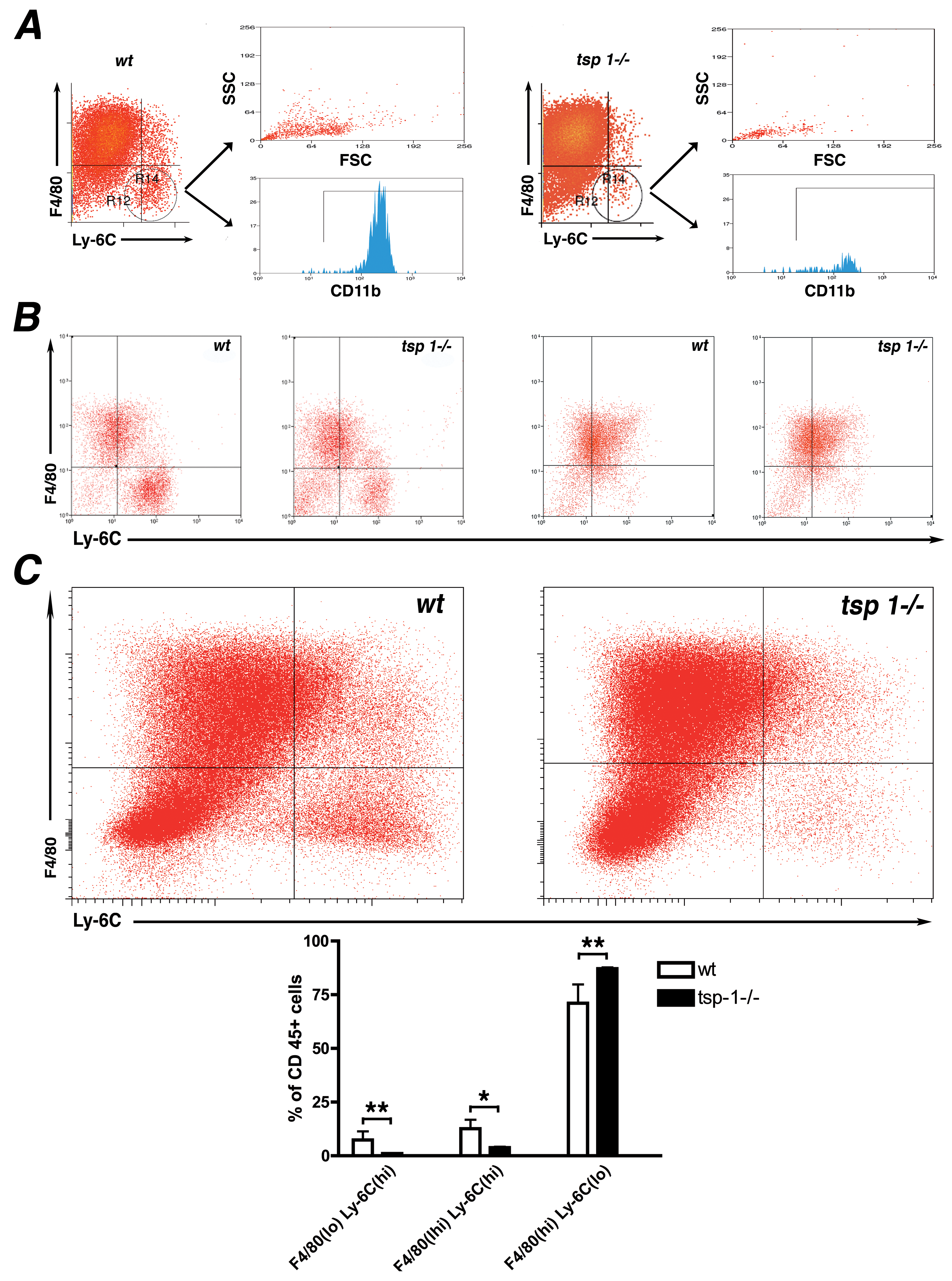

Supplement: Figure S4 — Thrombospondin-1−/− mice exhibit a less pro-inflammatory macrophage activation state in response to ischemia. Additional experiments. (A) SSC/FSC characteristics and CD11b expression of CD45+ F4/80(lo) Ly-6C(hi) cells isolated from ischemic muscles at d4, in both genotypes, showing an homogeneous SSC(lo)CD11b(hi) macrophage population. (B) FACS analyses of CD45+ cells isolated from ischemic muscles at d4, stained for F4/80 and Ly-6C expression. Additional examples of two independent experiments are shown (n = 5 mice). (C) Upper panel, representative FACS analyses of Ficoll-isolated mononuclear cells from ischemic muscles of one mouse from both genotypes at d4, stained for F4/80 and Ly-6C. Lower panel, quantification of F4/80(lo)Ly-6C(hi), F4/80(hi)Ly-6C(hi) and F4/80(hi)Ly-6C(lo) macrophage proportions in both genotypes (n = 5). (6.79 MB TIF) [file pone.0003950.s004.tif]
